# Assessment of coronary artery disease severity via non-invasive detection of *ABCA1* promoter methylation

**DOI:** 10.3389/fcvm.2026.1876840

**Published:** 2026-08-18

**Authors:** Liyun Peng, Ruowen Chen, Bin Chen

**Affiliations:** 1 Shengli Clinical Medical College of Fujian Medical University, Fuzhou, China; 2 Department of Cardiology, Cardiac Rehabilitation Center, Fuzhou University Affiliated Provincial Hospital, Fuzhou, China; 3 School of Public Health and Health Management, Fujian Health College, Fuzhou, China

**Keywords:** *ABCA1*, coronary artery disease, Gensini score, methylation, severity

## Abstract

**Background:**

*ABCA1* is a critical mediator of cholesterol efflux, and hypermethylation of its promoter may lead to transcriptional silencing. However, the relationship between *ABCA1* promoter methylation and the severity of coronary artery disease (CAD) remains poorly understood. This study aimed to explore whether *ABCA1* promoter methylation correlates with CAD severity and whether it could be used as a non-invasive epigenetic biomarker for auxiliary risk evaluation.

**Methods:**

This case-control study enrolled 80 consecutive patients with CAD (confirmed by coronary angiography) and 80 healthy controls from January 2025 to January 2026. *ABCA1* promoter methylation level was quantified using quantitative methylation-specific PCR. Associations with CAD diagnosis, Gensini scores, and clinical parameters were examined, with subgroup analyses by sex and age.

**Results:**

*ABCA1* methylation levels were significantly elevated in the patients with CAD compared with the controls [median (IQR): 14.01% (8.11–19.28) vs. 9.99% (5.91–13.45), *P* < 0.05]. Logistic regression revealed that hypermethylation was an independent epigenetic correlate of CAD (OR = 1.087 per 1% increase, 95% CI: 1.010–1.170). This multivariable model was fully adjusted for age, sex, BMI, and serum lipid profile [total cholesterol (TC), triglycerides, high-density lipoprotein cholesterol, low-density lipoprotein cholesterol (LDL-C), apolipoprotein A, and apolipoprotein B (ApoB)]. Among the patients with CAD, methylation level positively correlated with Gensini score (*r_s_* = 0.311, *P* < 0.05), TC (*r_s_* = 0.294, *P* < 0.05), LDL-C (*r_s_* = 0.284, *P* < 0.05), and ApoB (*r_s_* = 0.365, *P* < 0.01). In the overall cohort, methylation also correlated with ApoB (*r_s_* = 0.249, *P* < 0.05), age (*r_s_* = 0.297, *P* < 0.01), and female sex (*r_s_* = 0.803, *P* < 0.05).

**Conclusions:**

Peripheral blood *ABCA1* promoter methylation shows preliminary potential as a non-invasive epigenetic biomarker to assist auxiliary evaluation of coronary lesion severity, with particular promise in younger adults and women.

## 1 Introduction

Coronary artery disease (CAD) remains one of the leading causes of mortality and disability worldwide (1). Accurate assessment of disease severity is crucial for guiding treatment decisions and determining prognosis in patients with CAD. Currently, clinical evaluation of CAD severity primarily relies on imaging indices derived from coronary angiography, such as the Gensini and Syntax scores (2, 3). Although these methods provide direct visualization of coronary artery stenosis, they are invasive procedures with inherent drawbacks, including exposure to ionizing radiation, risk of contrast-induced nephropathy, vascular complications, and high operational costs (4, 5). In addition, serum biomarkers such as cardiac troponin I (cTnI) and brain natriuretic peptide are useful for assessing myocardial injury and cardiac function (6, 7). However, their specificity and sensitivity for detecting early-stage coronary lesions and evaluating overall atherosclerotic burden remain limited, making it difficult to comprehensively reflect the progression of disease severity. Therefore, there is an urgent clinical need to develop simpler, non-invasive, and dynamically monitorable approaches for CAD risk stratification.

DNA methylation, the most extensively studied epigenetic biomarker, enables reversible regulation of gene expression without changing the DNA sequence itself. Recent epigenome-wide association studies have identified differentially methylated regions associated with CAD risk, suggesting that locus-specific methylation patterns may serve as quantitative biological markers of disease severity (8, 9). ATP-binding cassette transporter A1 (*ABCA1*) is the rate-limiting step in the reverse cholesterol transport pathway and its impaired function could lead to macrophage cholesterol accumulation and atherosclerosis (AS) progression. Although genetic polymorphisms of *ABCA1* have been well-characterized (10), the epigenetic mechanism of functional silencing caused by promoter hypermethylation and its association with the severity of coronary lesions (Gensini score), as well as interactions with age and sex, remain insufficiently explored.

Therefore, the present study aimed to investigate the characteristics of *ABCA1* gene promoter methylation level in patients with CAD, analyze their correlation with the severity of coronary artery lesions (assessed by the Gensini score) and lipid metabolism parameters [particularly apolipoprotein B (ApoB) levels], and evaluate its correlative clinical potential as a non-invasive epigenetic biomarker for auxiliary assessment of CAD risk and lesion severity.

## 2 Methods

### 2.1 Participants

This study received approval from the Ethics Committee of Fuzhou University Affiliated Provincial Hospital (Approval No. K2022-09-091) and was carried out in compliance with the Declaration of Helsinki. Written informed consent was obtained from each participant. Between January 2025 and January 2026, we consecutively enrolled 80 patients with CAD, confirmed by coronary angiography, from the Department of Cardiology, Fuzhou University Affiliated Provincial Hospital, and 80 healthy individuals were recruited as controls. CAD was defined as the presence of ≥50% stenosis in at least one coronary artery or its major branches, as determined by Judkins coronary angiography. Cases with congenital heart disease; pulmonary, hypertensive, or rheumatic heart disease; severe hepatic or renal dysfunction; a history of malignancy; or who had received statin lipid-lowering therapy or other medications affecting lipid levels within the 3 months prior to enrollment were excluded.

### 2.2 Data collection

General demographic data were collected using questionnaires, including name, sex, age, body mass index (BMI), and relevant medical history. Fasting antecubital venous blood (5 mL) was collected on the second morning after admission, with 2 mL stored at −20℃ and the remaining 3 mL used to measure serum total cholesterol (TC), triglycerides (TG), high-density lipoprotein cholesterol (HDL-C), low-density lipoprotein cholesterol (LDL-C), apolipoprotein A (ApoA), and ApoB using enzymatic methods on a Siemens fully automatic biochemical analyzer (Siemens ADVIA 2400 Chemistry System, Germany). Gensini scores were calculated based on coronary angiography results (11).

### 2.3 DNA extraction

Genomic DNA was isolated by spin column-based purification according to the manufacturer's instructions. Briefly, after mixing 200 μL of peripheral blood with 20 μL of proteinase K and 200 μL of lysis GB (guanidinium binding buffer), the mixture was incubated at 70 °C for 10 min. The mixture was supplemented with 200 μL of absolute ethanol. It was then transferred to a CB3 adsorption column and spun at 12,000 rpm for 30 s. Next, the column underwent sequential washes: first with 500 μL of GD (guanidinium decontamination buffer), and then twice with 600 μL of wash PW (precipitation wash buffer). Finally, the column was centrifuged at 12,000 rpm for 2 min. After air-drying at room temperature, the adsorbed DNA was eluted in 50–200 μL of TE buffer (tris-EDTA buffer), characterized for concentration (50–100 ng/μL) and purity (A260/280 = 1.8–2.0) by UV spectrophotometry, and finally stored at −20 °C.

### 2.4 Bisulfite modification

We subjected 500–1,000 ng of genomic DNA to bisulfite conversion using a bisulfite conversion kit (DP304, Tiangen). The reaction system (120 μL) contained 20 μL DNA sample, 10 μL buffer DP, and 90 μL Bisulfite Mix. To drive the conversion to completion, the samples underwent a two-step incubation: first at 95 °C for 10 min, and then at 64 °C for 30 min. Under these conditions, unmethylated cytosines are converted to uracil, while methylated cytosines remain unmodified. Converted DNA was purified using adsorption column CB1 as follows: 600 μL binding PB (precipitation buffer) was added for binding, followed by two washes with 600 μL wash buffer PW, desulfonation with 600 μL solution DB (desorption buffer) under ambient conditions for 15 min, and two additional washes with 600 μL wash buffer PW. After centrifugation at 12,000 rpm for 2 min and subsequent air-drying, DNA elution was achieved by adding 20 μL of EB (elution buffer).

### 2.5 Quantitative methylation-specific PCR

A two-step fluorescence quantitative methylation-specific PCR (qMSP) was employed to quantify the methylation level of the *ABCA1* promoter region (GRCh38, chr9:107543000–107544200). All primer sequences were designed via an online bioinformatics tool, and full sequences of outer, methylated (M), and unmethylated (U) primers were listed in Table 1. The target amplicon spanned a 290 bp promoter fragment covering 12 cytosine-phosphate-guanine (CpG) dinucleotide sites, which are critical regulatory loci for *ABCA1* transcriptional activity. First-round PCR (pre-amplification) was as follows: 1 μL of genomic DNA was used as a template in a 20 μL reaction system containing 4 μL 5× Gotaq buffer, 1.6 μL dNTPs (2.5 mM), 0.4 μL each of *ABCA1* outer primers (10 μM), and 0.2 μL Gotaq enzyme (Promega, USA). The thermal cycling protocol began with an initial denaturation at 94 °C for 3 min. This was followed by 30 cycles, each consisting of denaturation at 94 °C for 30 s, annealing at 68 °C for 30 s, and extension at 72 °C for 1 min. A final extension step was then performed at 72 °C for 3 min. The second-round PCR (methylation-specific amplification) was as follows: The SYBR Green method was employed, with 1 μL of the product from the first-round PCR serving as the template (2× SYBR Master Mix, Kapa, KK4607) in a 10 μL system containing 0.8 μL M- or U-specific primer mixtures (5 μM each). The cycling protocol started with an initial denaturation at 98 °C for 2 min, followed by 40 cycles of 98 °C for 10 s and 58 °C for 30 s, with fluorescence signals acquired during the 58 °C step.

**Table 1:** Primer sequences for methylation-specific PCR.

Primer name	Primer type	Sequence (5'→3’)
*ABCA1*-out-F	Outer forward primer	GAAAGTAGGATTTAGAGGAAGTAAATTTTA
*ABCA1*-out-R	Outer reverse primer	AATTCAATCACTCAACAAAAAACAC
*ABCA1*-M-F	Methylated forward primer	AATTTTATTGGTGTTTTTGGTTGTC
*ABCA1*-M-R	Methylated reverse primer	ATATCTTAAAATCCGCGATCTACG
*ABCA1*-U-F	Unmethylated forward primer	AATTTTATTGGTGTTTTTGGTTGTT
*ABCA1*-U-R	Unmethylated reverse primer	TATCTTAAAATCCACAATCTACATC

M, methylated primer; U, unmethylated primer; F, forward; R, reverse.

After verifying amplification efficiency (approximately 2.0) through 10-fold gradient dilutions, relative quantification was analyzed using the 2^–△Ct^ method. The methylation index (MI) was calculated as MI = [M/(M + U)] × 100%, where M represents the methylated copy number and U represents the unmethylated copy number. Given that methylation levels vary substantially across distinct CpG loci within the *ABCA1* promoter, all quantitative data in the present study exclusively reflect the methylation status of the 12 CpG sites covered by the designed amplicons.

### 2.6 Statistical analysis

The statistical analyses were performed using SPSS 25.0. Continuous variables with normal distribution are shown as mean ± SD (x¯ ± s), while those with skewed distribution are shown as median (P25, P75) [M (P25, P75)]. Group comparisons were made using the independent t-test for normally distributed data with equal variances, and the Mann–Whitney *U* test for all other cases. For unordered categorical variables, the chi-square (*χ*^2^) test was applied for comparisons, with the data expressed as frequencies and percentages. When bivariate data followed a normal distribution, Pearson correlation analysis was performed; otherwise, Spearman's rank correlation coefficient (*r*ₛ) was used. Binary logistic regression analysis was used to identify independent epigenetic correlates of CAD. The covariates adjusted for in the multivariable model included age, sex, BMI, triglycerides, total cholesterol, LDL-C, HDL-C, ApoA and ApoB, which are well-established conventional cardiovascular risk factors. A *P*-value < 0.05 was considered statistically significant.

## 3 Results

### 3.1 Demographic characteristics

A total of 160 participants were enrolled in the present study, consisting of 80 patients with confirmed CAD and 80 healthy controls. As detailed in Table 2, the two groups were well-balanced with respect to baseline demographic and clinical characteristics. Specifically, no significant differences were observed between the CAD group and the control group in terms of sex distribution (60.0% vs. 56.25% male, *P* = 0.631), age [63.00 (55.00, 70.00) vs. 61.00 (52.00, 68.00) years, *P* = 0.468], or BMI [23.86 (21.95, 26.57) vs. 23.87 (22.35, 25.34) kg/m^2^, *P* = 0.917]. Furthermore, all measured lipid parameters, including TG, TC, HDL-C, LDL-C, ApoA, and ApoB, showed no significant intergroup differences, indicating that the two groups were comparable at baseline.

**Table 2:** Demographic characteristics of the participants.

Characteristic	Control group (*N* = 80)	CAD group (*N* = 80)	*t*/*Z*/*χ*^2^	*P-*value
Men (*n*, %)	45 (56.25)	48 (60.00)	0.231	0.631
Age (years)	61.00 (52.00,68.00)	63.00 (55.00,70.00)	−0.725	0.468
BMI (kg/m^2^)	23.87 (22.35,25.34)	23.86 (21.95,26.57)	−0.099	0.917
TG (mmol/L)	1.33 (1.04,2.02)	1.56 (1.20,2.01)	−1.133	0.257
TC (mmol/L)	4.72 ± 1.10	4.36 ± 1.19	−1.345	0.182
HDL-C (mmol/L)	1.28 ± 0.31	1.21 ± 0.26	−1.125	0.261
LDL-C (mmol/L)	3.32 ± 1.06	3.08 ± 1.15	−0.944	0.348
ApoA (g/L)	1.32 ± 0.22	1.28 ± 0.20	−0.969	0.333
ApoB (g/L)	1.00 ± 0.24	0.99 ± 0.26	−0.087	0.931

BMI, body mass index; TG, triglycerides; TC, total cholesterol; HDL-C, high-density lipoprotein cholesterol; LDL-C, low-density lipoprotein cholesterol; ApoA, apolipoprotein A; ApoB, apolipoprotein B.

### 3.2 Association between *ABCA1* gene promoter methylation level and coronary artery disease

As shown in Table 3, the CAD group exhibited a substantially higher *ABCA1* promoter methylation level compared with the healthy control group. The median methylation level in the patients with CAD was 14.01% (IQR: 8.11%–19.28%), whereas that in the controls was 9.99% (IQR: 5.91%–13.45%). This difference was statistically significant (Z = −2.341, *P* = 0.019), suggesting that hypermethylation of the *ABCA1* gene may be associated with CAD susceptibility.

**Table 3:** Comparison of *ABCA1* methylation level between patients with CAD and healthy controls.

Item	Control group	CAD group	*Z*	*P value*
*N*	80	80		
Methylation level (%)	9.99 (5.91,13.45)	14.01 (8.11,19.28)	−2.341	**0.019**

Bold *P* value indicates a statistically significant difference in *ABCA1* methylation levels between the CAD group and healthy control group (*P* < 0.05). Intergroup comparisons were conducted using the Mann–Whitney *U* test, with results reported as the *Z* statistic.

To further investigate whether the association between *ABCA1* methylation and CAD varied across different subgroups, we performed stratified analyses by sex and age. The results are summarized in Table 4. In terms of sex differences, female patients with CAD showed a markedly higher mean methylation level (15.36 ± 8.71%) compared with the female controls (10.08 ± 4.76%), with the difference reaching statistical significance (*t* = 2.123, *P* = 0.043). In contrast, although the male patients with CAD also exhibited elevated methylation levels relative to the male controls (14.74 ± 7.50% vs. 12.74 ± 8.76%), this difference did not achieve statistical significance (*t* = 0.730, *P* = 0.469).

**Table 4:** Stratified analysis of *ABCA1* methylation level by sex and age in patients with CAD and healthy controls.

Item	Gender		Age	
	Men	Women	<65 years	≥65 years
CAD group	14.74 ± 7.50	15.36 ± 8.71	14.33 ± 6.36	15.26 ± 8.60
Control group	12.74 ± 8.76	10.08 ± 4.76	10.23 ± 6.75	12.69 ± 5.24
*t*	0.730	2.123	2.051	0.843
*P-*value	0.469	**0.043**	**0.047**	0.405

Bold *P* values indicate statistically significant differences in *ABCA1* methylation levels between the CAD group and healthy control group (*P* < 0.05). Intergroup comparisons were conducted using the independent-samples *t*-test.

With regard to age stratification, we observed that among participants younger than 65 years, the patients with CAD had significantly higher *ABCA1* methylation levels than their healthy counterparts (14.33 ± 6.36% vs. 10.23 ± 6.75%, *t* = 2.051, *P* = 0.047). However, in the older age subgroup (≥65 years), although the patients with CAD still demonstrated numerically higher methylation levels (15.26 ± 8.60% vs. 12.69 ± 5.24%), the difference was not statistically significant (*t* = 0.843, *P* = 0.405).

### 3.3 Multifactorial association analysis of *ABCA1* gene promoter methylation level

#### 3.3.1 Correlation between *ABCA1* gene promoter methylation level and age

Among all the subjects, *ABCA1* methylation level was positively correlated with age (Spearman correlation analysis: *r_s_* = 0.297, *P* = 0.007, Table 5; linear regression equation: methylation level = 0.1930 × Age + 1.505, *R*^2^ = 0.09643). The sex-stratified analysis indicated that *ABCA1* methylation level was positively correlated with age in the female subjects (all female subjects, *r_s_* = 0.667, *P* < 0.01; CAD group, *r_s_* = 0.803, *P* < 0.05; control group, *r_s_* = 0.539, *P* < 0.05, Table 5).

**Table 5:** Correlation analysis of *ABCA1* methylation level with age in the CAD and control groups.

Gender	Control group (*N* = 80)		CAD group (*N* = 80)		All subjects (*N* = 160)	
	*r_s_*	*P*	*r_s_*	*P*	*r_s_*	*P*
Men	0.006	0.987	0.060	0.710	0.093	0.514
Women	0.539	**0**.**014**	0.803	**0**.**009**	0.667	**0**.**000**
Total	0.313	0.093	0.208	0.147	0.297	**0**.**007**

Bold *P* values indicate statistically significant positive correlations between *ABCA1* methylation levels and age (*P* < 0.05). *r*_*s*_ denotes Spearman's rank correlation coefficient used for correlation analysis.

#### 3.3.2 Correlation between *ABCA1* gene promoter methylation level and severity of coronary lesions

**Figure 1:**
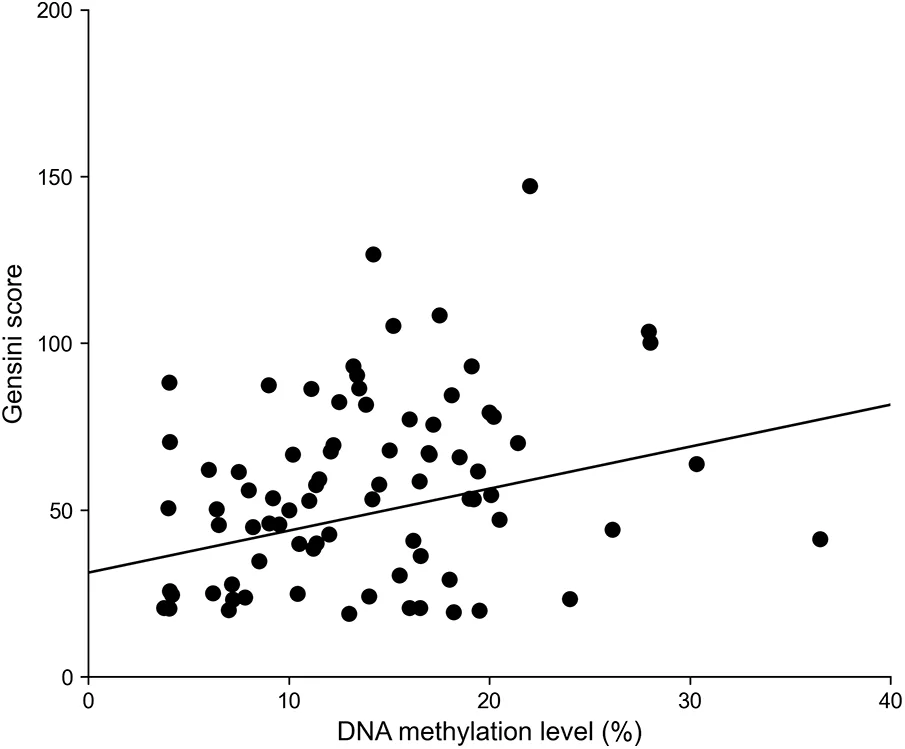
Correlation between *ABCA1* promoter methylation level and Gensini score in patients with CAD. The scatter plot shows the cross-sectional correlation between peripheral blood *ABCA1* promoter methylation percentage and Gensini score among 80 patients with angiographically confirmed CAD; Spearman *r_s_* = 0.311, *P* < 0.05; linear regression equation: Gensini score = 1.259 × methylation level (%) + 31.22.

To determine whether *ABCA1* methylation level correlates with the anatomical severity of coronary atherosclerosis, we analyzed the relationship between promoter methylation level and Gensini score among the 80 patients with CAD. The Spearman correlation analysis demonstrated a significant positive association (*r*ₛ = 0.311, *P* < 0.05), indicating that individuals with higher *ABCA1* methylation levels tended to have more extensive coronary artery lesions (Figure 1).

We further performed linear regression to quantify this relationship. The resulting equation, i.e., Gensini score = 1.259 × methylation level (%) + 31.22, suggests that for every 1% increase in *ABCA1* methylation, the Gensini score rises by approximately 1.26 points. Despite this modest explanatory power, the significant correlation observed supports the potential role of *ABCA1* methylation as a contributing factor to CAD severity.

#### 3.3.3 Correlation between *ABCA1* gene promoter methylation level and lipid profile

As summarized in Table 6, across all 160 subjects, *ABCA1* promoter methylation level showed a modest but statistically significant positive correlation with ApoB level (Spearman’s *r*ₛ = 0.249, *P* < 0.05), suggesting an association between *ABCA1* hypermethylation and increased circulating ApoB.

**Table 6:** Association of *ABCA1* methylation level with serum lipids.

Lipid parameter	Control group (*N* = 80)		CAD group (*N* = 80)		All subjects (*N* = 160)	
	*r_s_*	*P*	*r_s_*	*P*	*r_s_*	*P*
TG (mmol/L)	0.010	0.958	0.190	0.186	0.170	0.133
TC (mmol/L)	−0.071	0.711	0.294	**0**.**038**	0.123	0.277
HDL-C (mmol/L)	−0.078	0.681	0.039	0.789	−0.167	0.140
LDL-C (mmol/L)	−0.092	0.627	0.284	**0**.**043**	0.124	0.272
ApoA (g/L)	0.046	0.811	0.197	0.171	−0.032	0.775
ApoB (g/L)	0.016	0.934	0.365	**0**.**009**	0.249	**0**.**026**

Bold *P* values indicate statistically significant correlations between *ABCA1* methylation levels and serum lipid profiles (*P* < 0.05). *r*_*s*_ denotes Spearman's rank correlation coefficient for correlation analyses.

When we restricted the analysis to the CAD group, stronger and more extensive correlations emerged. Specifically, *ABCA1* methylation level in the patients with CAD was positively correlated with TC (*r*ₛ = 0.294, *P* < 0.05), LDL-C (*r*ₛ = 0.284, *P* < 0.05), and ApoB (*r*ₛ = 0.365, *P* < 0.01). Notably, the correlation with ApoB was particularly robust, exhibiting both the highest correlation coefficient and the lowest *P*-value among the three lipid parameters. These findings indicate that in patients with established CAD, elevated *ABCA1* promoter methylation is associated with a more atherogenic lipid profile.

## 4 Discussion

This study investigated the association between *ABCA1* promoter methylation level and CAD and its severity. We found that *ABCA1* hypermethylation was an independent epigenetic correlate of CAD but also exhibited a dose–response relationship with coronary lesion severity, demonstrating stronger correlations in female and young-onset CAD subgroups. These correlative observations indicate that peripheral blood *ABCA1* methylation may serve as a candidate non-invasive epigenetic biomarker to assist auxiliary CAD risk evaluation, with more prominent correlations observed in younger patients and women. Furthermore, this epigenetic marker may provide supplementary insights into residual cardiovascular risk independent of routine lipid indicators, and it represents a preliminary candidate locus for follow-up epigenetic intervention research aiming to restore reverse cholesterol transport capacity.

As the rate-limiting factor in reverse cholesterol transport, *ABCA1* dysfunction represents a central component in the pathogenesis and progression of AS (12). Previous studies have predominantly focused on associations between *ABCA1* genetic polymorphisms and CAD; however, static genetic variations cannot adequately explain dynamic gene-environment interactions (13). Furthermore, whether functional silencing of *ABCA1* expression originates from epigenetic modifications in the promoter region and its quantitative relationship with coronary lesion severity remain critical gaps in the field. Recently, DNA methylation, as a reversible epigenetic modification, has been established as a key bridge connecting metabolic stress to gene expression silencing (14). In this study, utilizing qMSP technology (15), we achieved precise quantification of *ABCA1* methylation level (expressed as percentages) and established, for the first time, a linear regression model with Gensini score (*R*^2^ = 0.061). We found that *ABCA1* methylation level was significantly positively correlated with Gensini score (*r_s_* = 0.311), suggesting that this epigenetic marker serves not merely as a discriminative indicator for CAD but potentially as a “functional silencing index” reflecting atherosclerotic plaque burden. This finding aligns with experimental studies by Brunham et al. (16) regarding the pivotal role of *ABCA1* in reverse cholesterol transport. Notably, although no significant differences were observed in conventional lipid parameters (TC, TG, LDL-C, and HDL-C) between the CAD and control groups in this study, *ABCA1* methylation level demonstrated a significant positive correlation with ApoB (*r_s_* = 0.365). This suggests that following achievement of standard lipid-lowering treatment targets, *ABCA1* promoter hypermethylation may drive residual cardiovascular risk by reducing cholesterol efflux capacity.

One of the most significant findings of this study is the robust correlation between *ABCA1* methylation and age, particularly prominent among the female patients with CAD (*r_s_* = 0.803), whereas no statistically significant correlation was observed in the male participants. This “sex–age–methylation” triple interaction effect reveals sexual dimorphism in CAD pathophysiology (17). Postmenopausal women experience a sudden increase in CAD risk that cannot be fully explained by traditional risk factors. While estrogen is believed to exert vascular protective effects by upregulating *ABCA1* expression to promote cholesterol efflux (18, 19), the molecular mechanisms governing *ABCA1* regulation following estrogen decline remain unclear. We hypothesize that estrogen withdrawal may accelerate vascular aging through epigenetic reprogramming. Under physiological conditions, estrogen maintains the low methylation status of the *ABCA1* promoter via estrogen receptor signaling activation, thereby promoting cholesterol efflux; however, following menopause, declining estrogen levels may lead to relatively enhanced DNA methyltransferase (DNMT) activity, coupled with accumulated oxidative stress, collectively driving *ABCA1* promoter hypermethylation. This finding provides a molecular explanation for the phenomenon of “accelerated vascular aging” in women (20). Additionally, this study revealed that the association between *ABCA1* methylation and CAD was statistically significant in the <65 years group but disappeared in the ≥65 years group, suggesting that *ABCA1* hypermethylation may serve as a specific biomarker for early-onset CAD. This age-stratified effect implies that in relatively younger patients, epigenetic dysregulation may represent a primary disease driver, whereas with advancing age, other cumulative damage (such as telomere shortening and inflammaging) may mask the independent effect of *ABCA1* methylation.

In the era of intensive lipid-lowering therapy, identifying molecular determinants of residual risk has become a critical challenge for cardiovascular disease prevention. In this study, the positive correlation between *ABCA1* methylation level and ApoB, independent of LDL-C, suggests its potential as a specific biomarker for residual cholesterol risk (21, 22). Compared to static lipid concentrations, *ABCA1* methylation level reflects the “functional capacity” of the reverse cholesterol transport pathway, more accurately capturing the dynamic progression of atherosclerosis. Although this study employed a cross-sectional design, the reversible nature of *ABCA1* methylation makes it a preliminary candidate marker worthy of further exploration for monitoring research. As emerging therapeutic targets for cardiovascular disease (23), epigenetic enzymes such as DNMTs hold significant drug development potential. Future research should explore restoring *ABCA1* expression through epigenetic intervention, implementing a “dual lipid-lowering” strategy (reducing LDL-C while simultaneously enhancing cholesterol efflux capacity) in combination with statin therapy. Previous studies have confirmed that peripheral blood DNA methylation signatures can independently and powerfully predict all-cause mortality (24). The qMSP technology employed in this study offers clinical accessibility, requiring only peripheral blood for detection—a simpler approach compared to invasive coronary angiography or expensive plaque imaging techniques—and thus providing a technical basis for follow-up research to establish auxiliary risk evaluation models based on *ABCA1* methylation levels.

Notably, owing to the cross-sectional design of the present study, we could not establish causal relationships between elevated *ABCA1* promoter methylation and the initiation or progression of coronary atherosclerosis. The observed positive correlations only reflect concurrent associations rather than causal effects, and longitudinal prospective studies are required to validate whether *ABCA1* methylation could predict future cardiovascular adverse events.

The limitations of this study include the relatively small sample size (80 patients with CAD and 80 healthy controls) and cross-sectional design, which preclude establishing causal relationships between *ABCA1* methylation and CAD progression. Although the sample size was appropriate for an initial exploratory case–control study, the stratified analyses by sex and age further reduced the sample size of each subgroup, which may have led to unstable effect size estimates and limited their statistical power to detect weak associations. Future validation in large prospective cohorts is required to confirm the predictive value of *ABCA1* methylation for cardiovascular events and to further clarify the direct impact of methylation modifications on *ABCA1* protein expression and cholesterol efflux function. Furthermore, sex-specific findings require replication in larger female cohorts, particularly with stratified analysis by menopausal status, to validate the hypothesis regarding estrogen-epigenetic interactions.

## 5 Conclusion

In conclusion, this cross-sectional study reveals significant cross-sectional correlations between *ABCA1* promoter hypermethylation and CAD diagnosis, coronary atherosclerotic lesion severity, and pro-atherogenic lipid markers (ApoB, TC, and LDL-C). Such correlative relationships were more prominent in the female patients and participants younger than 65 years. Collectively, peripheral blood *ABCA1* methylation exhibits preliminary potential as a non-invasive epigenetic biomarker for auxiliary evaluation of coronary lesion severity. The present findings provide foundational observational evidence for subsequent large prospective cohort validation and epigenetic functional research, which may further support precision cardiovascular research and individualized lipid regulation exploration in relatively younger and female populations.

## Data Availability

The original contributions presented in the study are included in the article/Supplementary Material, further inquiries can be directed to the corresponding author.
